# Behavioral Variant Frontotemporal Dementia in the Context of Progressive Apraxia of Speech: A Clinico-Neuroimaging Case–Control Study

**DOI:** 10.3390/brainsci15111169

**Published:** 2025-10-30

**Authors:** Nadia Hossain, Jerusha Bhaskaran, Joseph R. Duffy, Heather M. Clark, Mary M. Machulda, Dennis W. Dickson, Jennifer L. Whitwell, Keith A. Josephs

**Affiliations:** 1 Department of Neurology, Behavioral Neurology, and Movement Disorders, Mayo Clinic, College of Medicine, and Science, 200 1st Street S.W., Rochester, MN 55905, USA; hossain.nadia@mayo.edu (N.H.); jduffy@mayo.edu (J.R.D.); clark.heather1@mayo.edu (H.M.C.); 2 Department of Radiology, Mayo Clinic, Rochester, MN 55905, USA; bhaskaran.jerusha@mayo.edu (J.B.); whitwell.jennifer@mayo.edu (J.L.W.); 3 Department of Psychiatry and Psychology, Mayo Clinic, Rochester, MN 55905, USA; machulda.mary@mayo.edu; 4 Department of Neuroscience, Mayo Clinic, Jacksonville, FL 32224, USA; dickson.dennis@mayo.edu

**Keywords:** apraxia of speech, atrophy, bvFTD, MRI, SPM

## Abstract

**Objective:** Progressive apraxia of speech (PAOS) is a neurodegenerative syndrome characterized by impaired motor speech planning and programming, whereas behavioral variant frontotemporal dementia (bvFTD) is characterized by deviant behavioral (e.g., personality and social) features. Clinical and anatomic characteristics of bvFTD in the context of PAOS are understudied. **Methods:** We identified 12 participants with PAOS and features that were consistent with bvFTD at baseline or follow-up. Eleven completed a head MRI scan. We compared clinical features and anatomical patterns of atrophy in these 11 PAOS-bvFTD participants to 11 matched PAOS participants without bvFTD and 22 age- and sex-matched healthy controls. Statistical Parametric Mapping (SPM) was applied to visualize gray matter volume across both groups compared to controls and each other. Medians and 25th and 75th percentiles were assessed in patients and across groups; Fisher’s Exact Test and Mann–Whitney U tests were applied using BlueSky software, version 10.3.1-Pro. **Results**: As expected, PAOS-bvFTD participants performed worse on the Frontal Behavioral Inventory (median: 33/72 vs. 10/72), 20-item behavioral assessment scale (4.5/20 vs. 1.0/20), and the Neuropsychiatry inventory (4/36 vs. 1.5/36) compared to the PAOS group (*p* < 0.01 for all), with no differences in other demographic, neurological, or language tests. Seven of the eleven PAOS-bvFTD participants had bvFTD features develop within three years of symptom onset. The PAOS-bvFTD and PAOS groups showed volume loss in frontal lobe regions compared to controls, with PAOS-bvFTD participants having more prefrontal volume loss than PAOS participants. **Conclusions:** Behavioral features consistent with bvFTD can co-occur in patients with PAOS and are related to greater atrophy of the prefrontal cortex.

## 1. Introduction

Apraxia of speech (AOS) is a motor speech planning and programming disorder characterized by impaired articulation and prosody [1]. It is usually caused by stroke [2,3], but in recent years, it has been linked to several neurodegenerative diseases, including progressive supranuclear palsy (PSP), corticobasal degeneration (CBD), and Pick’s disease [3]. In neurodegenerative diseases, AOS may occur as part of complex symptoms, such as in the context of corticobasal syndrome (CBS) or the non-fluent/agrammatic variant of primary progressive aphasia (nfvPPA) [4]. AOS can also present as a relatively isolated or dominant symptom, without evidence of aphasia, where it has been referred to as primary progressive AOS (PPAOS) [4]. Collectively, these presentations are subsumed under the umbrella term of progressive apraxia of speech (PAOS) [5].

As a separate and distinct neurodegenerative syndrome, patients can present with progressive changes in comportment, where they are labeled as having a behavioral variant of frontotemporal dementia (bvFTD) [6]. Patients with bvFTD typically present at a young age (<65 years) [7] with behavioral and personality changes such as disinhibition, apathy, loss of empathy, compulsive behaviors, hyperorality, and neuropsychiatric behaviors. International consensus criteria recognize two categories of possible and probable bvFTD based on the number of symptoms present and whether there is supportive neuroimaging evidence of frontal and/or temporal lobe atrophy or dysfunction [8,9].

Clinical and neuroimaging aspects of PAOS and bvFTD have been studied extensively but separately. There is very little, if any, research on patients who have overlapping features of PAOS and bvFTD. Here, we aim to describe the clinical and neuroimaging features of patients with PAOS who also have or later develop features of bvFTD (PAOS-bvFTD) and to compare these with patients with PAOS who do not exhibit any features of bvFTD.

## 2. Methods

### 2.1. Protocol and Patient Consent

This study was approved by the Mayo Institutional Review Board (IRB), and all participants provided written informed consent for their information/data to be utilized for research purposes.

### 2.2. Participants and Recruitment Criteria

Patients with PAOS who presented to the Department of Neurology at the Mayo Clinic, MN, were recruited by the Neurodegenerative Research Group into a speech- and language-focused NIH-funded study between July 2010 and March 2025 and followed longitudinally over time. Inclusion criteria included AOS that was insidious at onset and progressive over time, with or without aphasia. A diagnosis of PAOS was based on consensus among at least two speech–language pathologists, as described in a previous study [10]. Given our aim to assess bvFTD in the context of PAOS, AOS must be present; participants with progressive agrammatic aphasia without AOS or those meeting criteria for probable CBS [11] or possible PSP–Speech/language variant [12] were excluded. To date, 124 participants with PAOS have been recruited. Of these, 12 have been noted by the evaluating neurologist (KAJ) to have some features of bvFTD based on features described in the FTD criteria by Neary and colleagues [6] at one or more time points. We will refer to these 12 patients as having PAOS-bvFTD. Of these 12 PAOS-bvFTD participants, all but one had completed an MRI brain scan. Hence, 11 PAOS-bvFTD participants were included in this study. Seven of the participants have since died, and four underwent a standardized autopsy by an expert neuropathologist (DWD), as previously described [13]. Two of the eleven PAOS-bvFTD participants have been previously described [14].

We matched these 11 PAOS-bvFTD participants with 11 “pure” PAOS participants who did not have any features of bvFTD or any other motor features at the time of presentation, and who had completed an identical MRI protocol. These 11 participants will be referred to as PAOS participants. The PAOS-bvFTD participants were matched by age, sex, AOS severity, AOS type, and aphasia severity to the PAOS participants. We also included a group of 22 healthy control participants for comparison. Individuals in the control group were matched 2:1 to the 11 PAOS-bvFTD participants by age and sex. None of the healthy control participants had any evidence of AOS, aphasia, cognitive, or motor impairment. Twelve (54.5%) were female, with a median age at scan of 61.8 years (IQR: 53.74, 72.36) and a median MoCA of 28.0 (IQR: 27.0, 29.0), and all had a Hoehn and Yahr score of 0.

### 2.3. Clinical Data

All participants underwent neurological assessments by a board-certified neurologist specializing in behavioral neurology, and all completed neuropsychological testing that was overseen by a board-certified neuropsychologist (MMM). The following validated clinical scales were completed in all patients and were included in the study: the Montreal Cognitive Assessment (MoCA) battery that assesses general cognition [15]; the Movement Disorder Society-Sponsored revision of the Unified Parkinson’s Disease Rating Scale III (MDS-UPDRS III) [16] for severity of motor Parkinsonism; the Western Aphasia Battery (WAB) praxis subtest [17] for severity of limb ideomotor apraxia and oral apraxia; the 15-item version of the Boston Naming Test (BNT) [18] for confrontation naming ability; the PSP saccadic impairment scale (PSIS) [19] for abnormalities in eye movement; the Incomplete Letters from the Visual Object and Space Perception Battery (VOSP) [20] to measure visuoperceptual ability; the Frontal Behavioral Inventory (FBI) [21] and the 20-item behavioral assessment scale (20-BAS) [22] to assess for behavioral disturbances; the Neuropsychiatric Inventory Questionnaire brief questionnaire (NPI-Q) [23,24] to evaluate and validate neuropsychiatric features [24]; the Frontal Assessment Battery (FAB) [25], Trail Making Test Part B (TMT-B) [26], and modified Wisconsin Card Sorting Test—Category Type (mWCST-CAT) [27] for executive dysfunction; and the PSP Rating Scale [28] to assess severity of features that have been associated with PSP, given that many PAOS patients have PSP pathology at death [29]. The Clinical Dementia Rating (CDR) [30,31], FBI [21], and 20-BAS [22] were all used to assess social and functional decline.

*Apolipoprotein E epsilon 4* genotype was assessed among participants, as described previously [32].

### 2.4. Speech and Language Data

Speech and language examinations of all 22 PAOS-bvFTD and PAOS participants in the study were performed by certified speech–language pathologists, with video and audio recordings being completed at the time of evaluation. These recordings were reviewed by study team members, including at least two board-certified speech–language pathologists, in a consensus meeting for discussion and diagnosis. The following speech and language variables were abstracted for this study: the Western Aphasia Battery—Revised aphasia quotient (WAB-AQ) [17], which also includes an animal fluency task to assess category fluency; letter fluency task (F) [33] to evaluate letter fluency; non-verbal oral apraxia (NVOA) testing [34] to assess the integrity of two trials each of four actions (“cough”, “click your tongue”, “blow”, “smack your lips”); and the AOS rating scale (ASRS-3) [35], including total score and phonetic and prosodic subscores to estimate the severity of AOS. Aphasia and AOS severity ratings (0 = absent; 4 = severe) were based on the judgment of the entire speech and language assessments by a team of speech–language pathologists [14].

### 2.5. Assessing bvFTD Criteria

For the 11 PAOS-bvFTD participants, we formally applied the bvFTD international criteria for probable and possible bvFTD [8] at each visit, with a modification to the term “disease duration”. We did not require that the criteria be met within three years from disease onset, given that the initial symptoms in our cohort were speech-related rather than core bvFTD features. We considered three major categories when applying the bvFTD criteria: Category 1, possible bvFTD; Category 2, probable bvFTD; and Category 3, exclusionary for bvFTD. To determine whether the bvFTD criteria were met, we abstracted symptoms from scales that focus on symptoms and signs related to bvFTD: the NPI-Q, FBI, 20-BAS score, VOSP, and the CDR. Signs and symptoms from these scales were also assessed as measures of disease severity and have been shown to correlate with patterns of cortical atrophy in bvFTD [36]. For participants who had features of bvFTD at the baseline research visit, we reviewed their history to determine the earliest onset of bvFTD features.

### 2.6. Neuroimaging Data

All 44 (11 + 11 + 22) participants, including controls, completed a volumetric 3.0T head MRI with a standard protocol [9], including a Magnetization-Prepared Rapid Gradient Echo (MPRAGE) sequence. Statistical Parametric Mapping (SPM) (www.fil.ion.ucl.ac.uk/spm, accessed on 24 April 2025) was used to compare gray matter volume loss between PAOS-bvFTD patients (using the scan at the visit when the participant first met modified bvFTD criteria), PAOS patients, and controls and was corrected for age and sex. All MPRAGE scans were normalized to the Mayo Clinic Adult Lifespan template (MCALT) and segmented using unified segmentation with MCALT priors and modulated, and the gray matter images were smoothed at 8mm full-width half maximum. Results were assessed at *p* < 0.001 uncorrected for multiple comparisons and at family-wise error (FWE) correction *p* < 0.05. The results were displayed on a brain render using BrainNet Viewer [37].

### 2.7. Statistical Analysis

We provide medians and 25th and 75th percentile (Q1, Q3) values for neurological, speech/language, and neuropsychiatric variables of interest across groups. To compare medians between the PAOS-bvFTD and PAOS groups, we used Fisher’s Exact Test for binary data and the Mann–Whitney U test for continuous data, with the assistance of BlueSky software, version 10.3.1-Pro, setting the significance level at *p* < 0.05.

## 3. Results

### 3.1. Demographic and Clinical Findings

The PAOS-bvFTD participants completed between one and four research visits. Table 1 provides detailed information on how each of the 11 PAOS-bvFTD participants met the modified bvFTD criteria longitudinally, with a modification for early symptom onset, as noted above. All 11 participants met the criteria for probable bvFTD.

Six participants met the bvFTD criteria at the first research visit, three at the second research visit, and two at the third research visit. Among the behavioral abnormalities, the most frequent were disinhibition (100%), apathy (90.9%), loss of sympathy or empathy (90.9%), and hyperorality (72.7%). Among the eleven PAOS-bvFTD participants, seven (64%) had onset of bvFTD features within the first three years of onset (participants 1, 2, 3, 5, 7, 8, and 11). The remaining four participants (36%) had an onset of bvFTD features after three years from onset. The median time from PAOS onset to fulfillment of the bvFTD criteria was 3 years.

Table 2 shows the demographic and clinical features of the 11 PAOS-bvFTD participants at the time they met the modified bvFTD criteria, compared to the matched PAOS group.

Six of the eleven PAOS-bvFTD participants (54.5%) were female. The median (interquartile range) age at onset was 57 (49, 69) years, and a trend was observed in the median time from symptom onset to baseline visit of 3.8 (2, 4) years. Eight participants had the phonetic AOS subtype, and two had the prosodic AOS subtype [38]. Seven participants had aphasia, which was, on average, mild in severity. As expected, performance on the FBI, NPI-Q, and 20-BAS tests was worse in PAOS-bvFTD patients compared to PAOS participants. The two groups did not differ in demographic features or in the severity of general cognition, motor, or speech and language testing.

Of the four PAOS-bvFTD participants who underwent autopsy, one had CBD, two had Pick’s disease, and one had a 3-repeat/4-repeat frontotemporal lobar degeneration tauopathy.

### 3.2. Neuroimaging Findings

**Figure 1:**
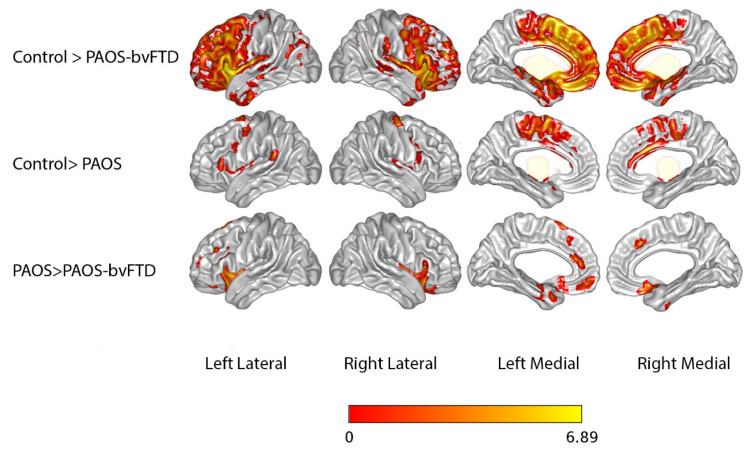
Brain renders across groups showing gray matter volume comparison.

Results from the SPM analysis comparing gray matter volume loss in PAOS-bvFTD participants, PAOS participants, and healthy controls are shown in Figure 1.

Figure 1 shows a voxel-based morphometry analysis of gray matter volume between controls and PAOS-bvFTD group (first row); controls and PAOS (middle row); and PAOS and PAOS-bvFTD (last row). Results are shown on lateral and medial three-dimensional renderings of the brain, which are uncorrected for multiple comparisons at *p* < 0.001 (bvFTD = behavioral variant frontotemporal dementia; PAOS = primary progressive apraxia of speech). Compared to controls, the PAOS-bvFTD group exhibited greater volume loss in the bilateral medial and lateral frontal lobes and the anterior–medial temporal region, with the findings being slightly worse in the left hemisphere. Findings in the posterior frontal lobes survived correction for multiple comparisons (Supplementary Figure S1). Compared to controls, the PAOS group showed focal volume loss in the bilateral supplementary motor cortex and lateral premotor cortex. The finding in the left supplementary motor area survived correction for multiple comparisons (Supplementary Figure S1). Compared to the PAOS group, the PAOS-bvFTD group showed greater volume loss, predominantly in the bilateral inferior and medial frontal lobes (Figure 1). The difference was present, uncorrected, at *p* < 0.001 but did not survive FWE corrections.

## 4. Discussion

In this study, we identified a cohort of participants with PAOS who developed behavioral features and met the modified criteria for probable bvFTD. The most common behavioral features were disinhibition, apathy, and loss of empathy/sympathy. These PAOS-bvFTD participants showed greater frontal lobe atrophy on MRI scans than PAOS participants without behavioral features who were matched for AOS and aphasia severity.

Seven of the eleven PAOS-bvFTD participants had behavioral changes that occurred very early in the disease, within three years of the onset of PAOS, demonstrating that behavioral changes can occur early in the disease course. Whether such patients are best considered as having bvFTD with PAOS, PAOS with bvFTD, or mixed bvFTD-PAOS is not clear. More specifically, it is unclear whether such patients should be considered to have a distinct syndrome versus two co-occurring syndromes, i.e., PAOS and bvFTD. It was less common for participants to develop bvFTD later in the disease course. In fact, of the four participants who developed bvFTD more than three years from onset, only one developed it more than five years from onset. Technically, these four participants did not meet stringent bvFTD criteria, given that behavioral changes developed after three years from the onset of speech symptoms; it is worth recognizing that the time course is based on patient and care partner reports, which might be a limitation when judging the relative onset of changes [8]. Modifying the bvFTD criteria, by ignoring the 3-year rule, allows for a diagnosis of bvFTD to be rendered in patients who later develop signs and symptoms that are consistent with bvFTD. Without such modification, a diagnosis of PAOS would be maintained, even in the presence of significant behavioral changes.

In the bvFTD criteria [8], the first criterion is “disinhibition”, defined as either socially inappropriate behavior, loss of manners, or impulsive or restless actions. In our PAOS-bvFTD participants, all 11 patients displayed at least one, if not all three of these features. This would be consistent with disinhibition being the most common feature in bvFTD [39]. The following two most frequent features in our PAOS-bvFTD participants were apathy and loss of sympathy or empathy, which is also consistent with findings from other studies on bvFTD [8,40]. Our PAOS-bvFTD participants had less frequent stereotyped or ritualistic behavior, which is also considered less frequent in bvFTD [39,41]. These results suggest that features of bvFTD in our PAOS participants are typical of the features that are most frequently observed in classic bvFTD. Interestingly, the bvFTD-PAOS participants did not perform more poorly on tests of executive function compared to the PAOS participants. One likely explanation for the lack of a difference, given that most bvFTD-PAOS patients had executive dysfunction, is that PAOS patients can have mild executive deficits [37], which would make it challenging to find significance with our sample size. Neuropsychological features are one of the six criteria needed for diagnosing bvFTD. In our bvFTD-PAOS participants, approximately three-quarters had neuropsychological features that were similar to those reported in a bvFTD criteria study (68%) [8]. This was due to more than 90% of our bvFTD-PAOS participants having executive dysfunction and relative sparing of episodic memory and visuospatial skills compared to executive function.

Behavioral variant FTD is associated with atrophy of the frontal lobes, especially the medial frontal lobes, and the anterior temporal lobes [42]. Regarding our neuroimaging findings, there was greater volume loss in the PAOS-bvFTD participants in both the lateral and medial frontal lobes, as well as in the bilateral anterior medial temporal lobes, compared to controls. This pattern is similar to that expected in bvFTD. In such instances, bvFTD cases have been labeled as having the frontal-dominant subtype of bvFTD [9]. When the PAOS-bvFTD participants were compared to the PAOS participants, there was greater inferior and medial frontal lobe atrophy, especially on the left, in the PAOS-bvFTD group. This finding would explain why PAOS-bvFTD participants exhibited more behavioral dyscontrol at the time they first met the modified bvFTD criteria. Both groups showed involvement of the medial and lateral premotor cortex, which we have previously shown is associated with AOS [3,43], and Broca’s area, which is related to agrammatic aphasia [43].

Several scales of behavioral change were evaluated in the current study. The FBI is a care partner-oriented questionnaire that assesses patients’ behavior by evaluating 24 different behavioral changes related to the frontal lobe, on a scale from 0 to 4 (0 = absent; 4 = severe) [21]. The NPI-Q assesses 12 behavioral features of a patient experienced by the caregiver, which are further subdivided as behavioral (disinhibition, apathy), appetite, or eating behavior; mood changes (anxiety, euphoria, irritability, depression); and disruptive or psychotic symptoms (delusion, agitation/aggression, night-time behavior), with a severity score ranging from 1 to 3 (1 = mild; 3 = severe) and is valid as a test for neuropsychiatric assessment [23,24]. The 20-BAS captures a broader range of behavioral and neuropsychiatric features, many of which the FBI or NPI-Q [22] do not assess. Thus, all these scales focus on symptoms that are pertinent to bvFTD. As expected, the PAOS-bvFTD participants performed worse on all three tests compared to the PAOS participants. Given this finding, we strongly suggest that at least one test assessing behavioral change be administered to patients presenting with PAOS. Further, we hypothesize that a “high” score on such a test at baseline evaluation may be indicative of co-occurring bvFTD or that features of bvFTD will develop over time.

There was a trend for a shorter disease duration in the PAOS-bvFTD group compared to the PAOS group. This will require validation in a larger cohort. Still, it could be that PAOS subjects who rapidly develop some behavioral symptoms and eventually meet a bvFTD diagnosis have a more rapid course and therefore present earlier to clinical attention. In contrast, those with isolated PAOS may have a more indolent course and thus longer disease duration at the time of evaluation.

Of the PAOS-bvFTD participants, seven had died, and four had undergone an autopsy evaluation. The most common pathology was Pick’s disease, which was found in 2/4 (50%) of cases. One participant (25%) had a 4-repeat tauopathy, CBD, and another had a pathology characterized by both 3-repeat and 4-repeat inclusions in frontotemporal regions. Of our previously published larger autopsy cohort of PAOS patients, all patients had a 4R tauopathy, with 17/27 (63%) having CBD and the remaining 37% having PSP [13]. We have previously shown that Pick’s disease is associated with PAOS in the context of other neurological signs and symptoms [13], including bvFTD features [44]. Detailed information on the two Pick’s disease patients has been previously reported [44]. We do wonder whether Pick’s disease may be more common in patients with PAOS-bvFTD, which would be intriguing if proven, given that 4R tauopathies are more common in PAOS patients without bvFTD. Furthermore, our findings may suggest that PSP pathology is unlikely to be associated with a syndrome characterized by both PAOS and bvFTD. Although we are not 100% certain about the underlying pathological mechanism that accounts for bvFTD-PAOS, it is possibly related to underlying pathologies that target both the prefrontal and premotor cortices, such as Pick’s, CBD, and FTLD-mixed 3R +4R tau. We noted that PSP pathology, which tends to relatively spare the prefrontal cortex, was not present in any of the bvFTD-PAOS cases.

It is notable that the majority (73%) of the PAOS-bvFTD patients had the phonetic subtype of AOS. This suggests that there may be an association between phonetic AOS and the development of behavioral features in PAOS, as the prosodic subtype is three times more likely to occur than the phonetic subtype in PAOS patients [38]. The phonetic subtype of AOS has been associated with the development of aphasia in patients with primary progressive apraxia of speech [38]. Further studies are needed to assess the relationship between behavioral features, aphasia, and phonetic AOS in order to understand this relationship better. The AOS subtype was matched between the PAOS-bvFTD and PAOS groups to avoid any confounds in the imaging analysis. We have previously found that prosodic AOS is more common than phonetic AOS in patients with PSP pathology, while phonetic AOS is more common in those with CBD pathology [13]. The phonetic subtype may also be associated with Pick’s disease. In fact, we have previously reported that one other PAOS patient with Pick’s disease had phonetic AOS [44].

Strengths of the current study include that all our participants were prospectively followed, and that data were collected from the baseline up until their last available visit. Diagnoses were made through consensus among an expert team using validated, standardized tests and procedures. The neurobehavioral features in our participants were evaluated using different tests rather than relying on a single test, which makes our findings more definitive. Nonetheless, our cohort was relatively small, and hence, the absence of other differences between groups could have been due to limited power to detect more subtle differences. A larger prospective study is needed to validate and support the generalizability of our findings and should include a bvFTD group without PAOS, which may help determine whether bvFTD-PAOS is a distinct entity or not. Furthermore, performance on the FBI was confounded by speech and language abnormalities, and performance on the WAB praxis test may have been influenced by non-verbal oral apraxia. Our cohort was limited to patients with PAOS, and hence, our findings may not generalize to the agrammatic variant of primary progressive aphasia where AOS is absent. In addition, we did not include PAOS participants who only exhibited subtle–mild behavioral changes or executive dysfunction but did not have enough features to meet the bvFTD criteria. We have labeled such patients as having +AOS syndrome [13]. Lastly, we acknowledge that the direct comparisons on VBM did not survive FWE correction.

## 5. Conclusions

We show that behavioral changes occur in some patients with PAOS early in the disease course, associated with greater involvement of the frontal lobe. Ongoing monitoring of behavioral changes is recommended in patients who present with PAOS. Future work should evaluate the appropriateness of overlapping nomenclature for patients with hybrid presentations of PAOS and bvFTD.

## Figures and Tables

**Table 1:** Behavioral variant frontotemporal dementia modified criteria.

Criteria	P1	P2	P3	P4	P5	P6	P7	P8	P9	P10	P11	Median or %
Time from PAOS onset to when bvFTD criteria were met (years)	3 *	1 *	2	5	1	4	2 *	3	4	9	2	3
Visit no. when bvFTD criteria were met	1	1	1	1	1	2	3	2	2	3	1	1
Possible bvFTD (any 3 from 1 to 6 should be met)												
1. Disinhibition	+	+	+	+	+	+	+	+	+	+	+	100%
2. Apathy	+	+	+	+	+	−	+	+	+	+	+	90.9%
3. Loss of empathy/sympathy	+	+	+	+	−	+	+	+	+	+	+	90.9%
4. Stereotyped behavior	−	+	+	+	−	−	+	−	+	+	+	63.6%
5. Hyperorality	+	+	−	+	−	+	+	+	+	+	−	72.7%
6. Neuropsychological features (6a–6c should all be met)	+	−	+	+	+	+	+	−	−	+	+	72.7%
6a. Impaired executive function	+	+	+	+	+	+	+	−	+	+	+	90.9%
6b. Relative sparing of episodic memory	+	−	+	+	+	+	+	+	+	+	+	90.9%
6c. Relative sparing of visuospatial function	+	+	+	+	+	+	+	+	−	+	+	90.9%
Probable bvFTD (all 3 should be met)												
7. Meets possible bvFTD criteria	+	+	+	+	+	+	+	+	+	+	+	100%
8. Exhibits significant social decline	+	+	+	+	+	+	+	+	+	+	+	100%
9. Imaging findings (frontal/temporal atrophy or hypometabolism)	+	+	+	+	+	+	+	+	+	+	+	100%
Exclusionary Criteria (all 3 should be (−) for probable)												
10. Other prominent non-degenerative or medical disorders	−	−	−	−	−	−	−	−	−	−	−	100%
11. Pronounced behavioral disturbances from other psychiatric disorders	−	−	−	−	−	−	−	−	−	−	−	100%
12. Biomarkers for Alzheimer’s or other degenerative diseases	−	−	−	−	−	−	−	−	−	−	−	100%

* Participants’ onset of behavioral/personality changes was determined using patient and caregiver reports. “+”, criteria present; “−“, criteria absent.

**Table 2:** Characteristics of PAOS-bvFTD participants at time of bvFTD diagnosis compared to the PAOS group.

Variable		PAOS-bvFTD (N = 11)	PAOS (N = 11)	*p*-Value
Demographic features				
Gender: Female		6 (55%)	6 (55%)	0.99
Race: White		11 (100%)	10 (90.9%)	0.31
Ethnicity: Hispanic or Latino		0 (0%)	0 (0%)	0.99
Handedness: Right		11 (100%)	9 (81.8)	0.14
Education (years)		16 (13, 17)	16 (13, 17)	0.89
Apo E4 (+)		2 (22%)	3 (33%)	0.60
Age at Visit, years		62 (53, 73)	64 (57, 71)	0.87
Age at Onset, years		57 (49, 69)	59 (50, 66)	0.97
Time from Onset to Visit (years)		3.8 (2.7, 4.2)	5.2 (4.7, 6.5)	0.06
Neurological and neuropsychological testing				
MoCA (/30)		19.0 (15.5, 25.5)	24.0 (20.0, 25.0)	0.13
NPI-Q (/36)		4.0 (3.5, 10.0)	1.5 (1.0, 2.8)	<0.01
20-BAS (/20)		4.5 (3.3, 5.0)	1.0 (0.0, 1.0)	<0.01
Frontal Behavioral Inventory (/72)		33.0 (29.0, 35.5)	10.0 (8.0, 17.0)	<0.01
Frontal Assessment Battery (/18)		13.0 (6.0, 16.5)	16.0 (13.0, 17.0)	0.26
MDS-UPDRS III (/120)		10.0 (5.0, 11.5)	11.0 (8.5, 18.0)	0.13
WAB-Praxis (/60)		57.0 (42.5, 58.5)	58.0 (54.5, 59.0)	0.41
PSPRS (/100)		12.0 (7.3, 14.5)	14.0 (7.8, 19.0)	0.52
PSIS (/5)		0.0 (0.0, 1.0)	1.0 (0.0, 1.0)	0.55
TMT-B		101.0 (65.0, 205.0)	251.0 (94.0, 300.0)	0.19
mWCST-CAT		22.0 (19.0, 55.0)	37.5 (32.3, 49.8)	0.32
VOSP Letters (/20)		20 (20.0, 20.0)	20 (20.0, 20.0)	0.99
Speech and language testing				
Boston Naming Test (/15)		14.0 (12.0, 14.0)	13.0 (12.0, 14.8)	0.97
Letter Fluency (f) (/27)		5.0 (1.5, 5.5)	6.0 (4.5, 10.0)	0.15
WAB-AQ (/100)		91.5 (81.3, 96.2)	94.2 (90.8, 96.2)	0.49
WAB Animal Fluency (/20)		10.0 (7.0, 19.0)	15.5 (10.5, 17.0)	0.46
ASRS-3 Total (/52)		23.0 (19.5, 24.5)	25.0 (18.5, 30.0)	0.51
ASRS-3 Phonetic Subscore (/16)		8.0 (6.5, 9.3)	12.0 (6.0, 14.0)	0.25
ASRS-3 Prosodic Subscore (/16)		7.0 (6.5, 8.5)	6.0 (4.5, 10.0)	0.65
NVOA (/32)		23.0 (11.0, 30.0)	27.5 (24.0, 30.0)	0.37
AOS type:	Mixed	1 (9.1%)	1 (9.1%)	0.82
	Phonetic	8 (72.7%)	9 (81.8%)	
	Prosodic	2 (18.2%)	1 (9.1%)	
AOS Severity (/4)		2.0 (1.8, 3.0)	2.3 (1.3, 3.4)	0.86
Aphasia Present, N (%)		7 (64%)	7 (64%)	0.99
Aphasia Severity (/4)		1.0 (1.0, 2.0)	1.0 (0.25, 1.0)	0.21

MoCA = The Montreal Cognitive Assessment; MDS-UPDRS III = Movement Disorder Society-Sponsored revision of the Unified Parkinson’s Disease Rating Scale III; WAB-Praxis = Western Aphasia Battery praxis subtest; PSPRS = Progressive Supranuclear Palsy Rating Scale; PSIS = progressive supranuclear palsy (PSP) saccadic impairment scale; NPI-Q = Neuropsychiatric Inventory Questionnaire brief questionnaire; 20-BAS = 20-item behavioral assessment; TMT-B = Trail Making Test Part B; mWCST-CAT = modified Wisconsin Card Sorting Test—Category Type; WAB-AQ = Western Aphasia Battery revised—Aphasia Quotient; ASRS-3 = Apraxia of Speech Rating Scale (Version 3); NVOA = non-verbal oral apraxia; VOSP letters = Visual Object and Space Perception Battery Incomplete Letter.

## Data Availability

Research data is available upon request to the corresponding author. The data that was utilized for this study is part of a large data set that will be made publicly available at the end of the NIH-funded grant in 2028.

## Supplementary Materials

The following supporting information can be downloaded at: https://www.mdpi.com/article/10.3390/brainsci15111169/s1, Figure S1: Brain renders showing regions of gray matter volume comparison between controls and PAOS-bvFTD (first row) and controls and PAOS (second row).
