# Supplementary material for: Behavioral Variant Frontotemporal Dementia in the Context of Progressive Apraxia of Speech: A Clinico-Neuroimaging Case–Control Study
Source: Brain Sci. 2025 Oct 30;15(11):1169. doi: 10.3390/brainsci15111169 (PMC12650068; doi:10.3390/brainsci15111169)
Supplement: Supplementary file 1 [file brainsci-15-01169-s001.zip › Supplementary Figure S1.pdf]

Supplementary Figure S1

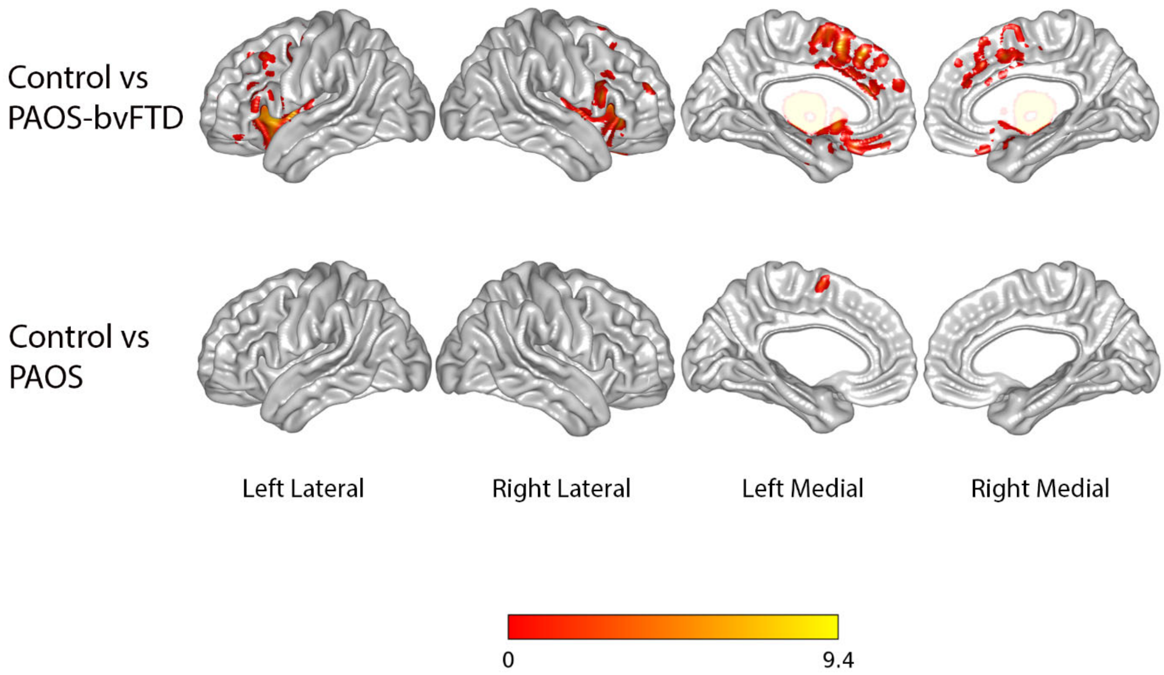

Supplementary Figure S1

Figure S1: Brain renders showing regions of gray matter volume comparison between controls and PAOS-bvFTD (first row) and controls and PAOS (second row). Results show lateral and medial 3-dimensional renderings of the brain, with significant differences after family wise error correction at p<0.05. bvFTD= behavioral variant frontotemporal dementia; PAOS= primary progressive apraxia of speech.
